# Mutation-informed gene pairs to predict melanoma metastasis

**DOI:** 10.1186/s12964-025-02602-4

**Published:** 2026-01-29

**Authors:** Seongsu Lim, Younggyun Lim, Ju Han Kim

**Affiliations:** Seoul National University Biomedical Informatics (SNUBI), Department of Biomedical Sciences, Seoul National University College of Medicine, Seoul, Republic of Korea

**Keywords:** Melanoma metastasis, Multi-Omics, Precision oncology, Drug repurposing

## Abstract

**Background:**

Metastasis causes over 90% of cancer-related deaths, including melanoma. However, most anti-cancer treatments focus on reducing tumor size rather than preventing metastatic spread. Therefore, there is a need to identify robust biomarkers that can predict and inhibit metastatic progression without inducing tumor cell death.

**Methods:**

We introduce the novel concept of synthetic anti-metastasis (SAM), which builds on the idea of synthetic lethality (SL). SAM pairs are interactions whose simultaneous impairment suppresses metastasis without inducing cell death. We identified preliminary SAM pairs using somatic mutation and clinical data from The Cancer Genome Atlas (TCGA). We selected the final SAM pairs by excluding previously reported SL interactions and pairs having at least one essential gene from preliminary pairs. We validated these SAM pairs across multiple datasets and tested their clinical relevance using survival analysis and machine learning (ML). Candidate anti-metastatic drugs for melanoma were identified through LINCS-based gene signature analysis, network analysis, and literature review.

**Results:**

We identified 325 final SAM pairs from 367 preliminary pairs. We found that patients with a high number of co-impairment or -inactivation of SAM pairs showed improved overall survival and reduced metastasis. The ML model based on SAM gene features accurately distinguished primary from metastases melanoma samples (AUROC: 0.940; HR: 0.724), outperforming models built from other known melanoma metastasis-associated genes. Finally, we discovered five compounds - MLN2480, pifithrin-µ, RO4929097, trametinib, and sorafenib - as potential anti-metastatic drugs for melanoma.

**Conclusions:**

This study provides SAM pairs as a novel type of biomarkers that could predict metastatic melanoma prognosis and as therapeutic targets in terms of reducing metastasis risk. Our framework to identify SAM pairs could offer a data-driven strategy to improve the prediction and discover potential treatment for melanoma metastasis through integrated genomic, transcriptomic, and pharmacogenomic analysis.

**Supplementary Information:**

The online version contains supplementary material available at 10.1186/s12964-025-02602-4.

## Introduction

In 2022, cancer was responsible for over 10 million deaths globally, with metastasis accounting for most of these fatalities [1–4]. Metastatic cancers, including melanoma, often change their location and functional characteristics, making them difficult to treat and strongly associated with poor prognosis [5–7]. However, most anti-cancer treatments have been designed by reducing tumor size rather than suppressing metastatic progression, which reflects a limited understanding of the molecular drivers of metastasis [6, 8].

Several studies have reported candidate biomarkers related to melanoma metastasis, based on biological datasets. Researchers represented these biomarkers not only as prognostic tools but also as potential therapeutic targets to reduce metastatic potential [9–13]. However, most of these studies relied on a limited number of transcriptomic datasets, resulting in their finding lacking robustness across various datasets, including mutation datasets. Also, some studies found some anti-tumor drugs linked to metastasis-related biomarkers, but did not aim to discover compounds having metastasis suppressive effects [9, 10]. Therefore, there is a need to identify additional biomarkers that could be used across various datasets and inform the discovery of novel anti-metastatic therapies for melanoma.

Synthetic lethality (SL) is a genetic interaction where only the simultaneous impairment of two genes results in cell death, while a cell lives when either gene or neither gene is impaired [14]. Researchers have induced selective cancer cell death without affecting normal cells by leveraging SL in oncology. For example, previous studies have demonstrated that SL can play an important role in precision medicine by offering potential opportunities as therapeutic target candidates or predictors of anti-cancer drug response [15–20]. Although these approaches have contributed to precision oncology, they primarily focus on tumor cell death but are not designed to address the process of metastasis. Moreover, SL interactions can be difficult to observe in tumor samples, since cancer cells with both genes impaired may already be eliminated through apoptosis. Also, current clinical strategies lack effective treatments that specifically target invasion or metastasis, even though these processes account for over 90% of deaths in solid tumors [8]. Therefore, the development of anti-cancer strategies based on SL-extended ideas could have the potential to provide significant breakthroughs in precision oncology.

To address this gap, we introduce the novel relationship, synthetic anti-metastasis (SAM), extended from SL: When two genes in the SAM interaction are damaged simultaneously, it does not induce melanoma cell death but suppresses metastatic progression. However, if either gene is damaged individually or not, metastasis will not be suppressed, leading to the death of the patient. This interaction may provide new opportunities to prevent metastasis-related mortality by inhibiting cancer cell migration or dissemination without causing toxicity through cell death.

In this study, we present a computational framework to identify SAM pairs in melanoma from various types of datasets. We validated their relevance by analyzing patient survival and tumor sample types across multiple datasets and approaches, including immune cell infiltration analysis, single-cell RNA sequencing analysis, functional enrichment analysis, and machine learning. Finally, we discovered candidate anti-metastatic compounds based on expression signatures of genes in SAM pairs and connectivity analysis. In summary, our results reveal a novel class of biomarkers that not only predict metastatic melanoma prognosis but may also help guide the development of therapies to reduce metastasis risk in melanoma.

## Results

### Overview of SAM research

**Fig. 1:**
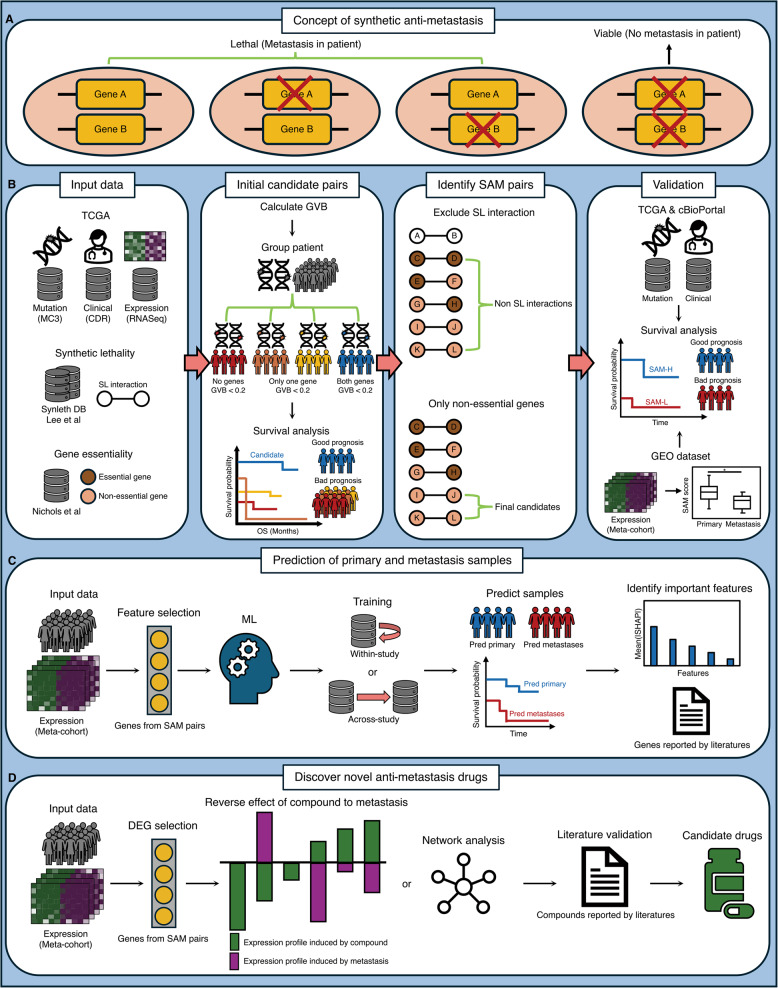
Research Overview. **A** A concept of a synthetic anti-metastasis (SAM) pair. **B** Overall pipeline to identify and validate SAM pairs (see “Methods”). **C** A machine learning (ML) approach based on genes in SAM pairs to predict primary and metastases samples from transcriptomic Meta-cohorts. **D** Discover candidate anti-metastasis drugs for melanoma using expression signatures of genes in SAM pairs

We conceptualized SAM interactions associated with the prediction and inhibition of metastasis in skin cutaneous melanoma (SKCM) based on SL (Fig. 1A).

We first identified preliminary SAM pairs using somatic mutation data from the TCGA MC3 project and corresponding clinical annotations from the TCGA Clinical Data Resource (CDR) for SKCM patients (Fig. 1B; See “Methods”) [21, 22]. The gene pairs of interest were those where patients with both impaired genes had better survival outcomes compared to those without such impairments. We quantified gene impairment using Gene-wise Variant Burden (GVB), with a score below 0.2 considered impaired (Supplementary Fig. 1; Supplementary Data S1; see “Methods”) [23]. To evaluate associations with survival using Cox proportional hazards regression, we stratified the melanoma patients into four groups based on the GVB status of each gene pair (Fig 1B; see “Methods”). 

To identify precise SAM gene pairs, we applied two filtering criteria to exclude gene pairs that were unlikely to reflect reducing metastasis risk (Fig. 1B; see “Methods”). First, we removed the previously identified SL gene pairs, as we aimed to avoid interactions associated with cancer cell death. Second, we excluded preliminary SAM pairs involving at least one essential gene, where impairment could result in cytotoxicity rather than predict metastasis or reduce metastasis risk.

Next, we evaluated whether these SAM pairs could stratify patients to predict cancer metastasis and survival outcomes (Fig. 1B; see “Methods”). In the validation process, we tested two hypotheses: (i) The patients with a high number of co-impaired or -inactivated SAM pairs would demonstrate better survival outcomes than who are not. (ii) The primary tumor samples would exhibit more co-impaired or -inactivated SAM pairs than metastases tumor samples. Therefore, we performed an internal validation using the original TCGA-SKCM somatic mutation and clinical data. We also performed an external validations using various independent somatic mutation datasets from cBioPortal and transcriptome datasets from the Gene Expression Omnibus (GEO).

To assess predictive utility of genes in SAM pairs, we trained machine learning (ML) models using expression signatures of genes in SAM pairs whether the models could accurately classify tumor samples as primary or metastases based on within- and across-study predictions (Fig. 1C; see “Methods”). These models were benchmarked against 11 other models based on previously published melanoma metastasis-related gene signatures (Supplementary Data S9). We then identified genes that significantly influenced to prediction performance in the ML model trained with genes in SAM pairs as features, using Shapley Additive exPlanation (SHAP) [23]. We also reviewed the existing literature to investigate whether these genes were associated with metastasis, especially in melanoma.

 Finally, we explored whether new candidate anti-metastasis compounds for melanoma could be discovered based on the genes in SAM pairs (Fig. 1D; see “Methods”). We performed drug repurposing based on iLINCS to identify potential anti-metastasis compounds for melanoma using the expression signatures of genes in SAM pairs from the meta-cohort used in ML [24]. Then, we investigated whether these compounds had anti-metastasis effects in melanoma using network analysis and literature searching.

### Identification of SAM interactions in TCGA-SKCM

We identified preliminary 367 SAM pairs based on somatic mutation and clinical data from TCGA-SKCM (see “Methods”). To identify precise pairs, we applied two filtering criteria to assess whether preliminary pairs met all required conditions (see “Methods”). We applied the first criterion, whether preliminary pairs were not experimentally or computationally identified as SL pairs. We found that 366 preliminary SAM pairs met the first criterion. We also applied the second criterion, whether the pair includes only non-essential genes. We found that 326 preliminary SAM pairs met the second criterion. As a result, we identified 325 SAM gene pairs that satisfied both criteria to use downstream validation and analysis (Supplementary Data S2).

### Internal validation for SAM pairs using the genomic dataset from TCGA

**Fig. 2:**
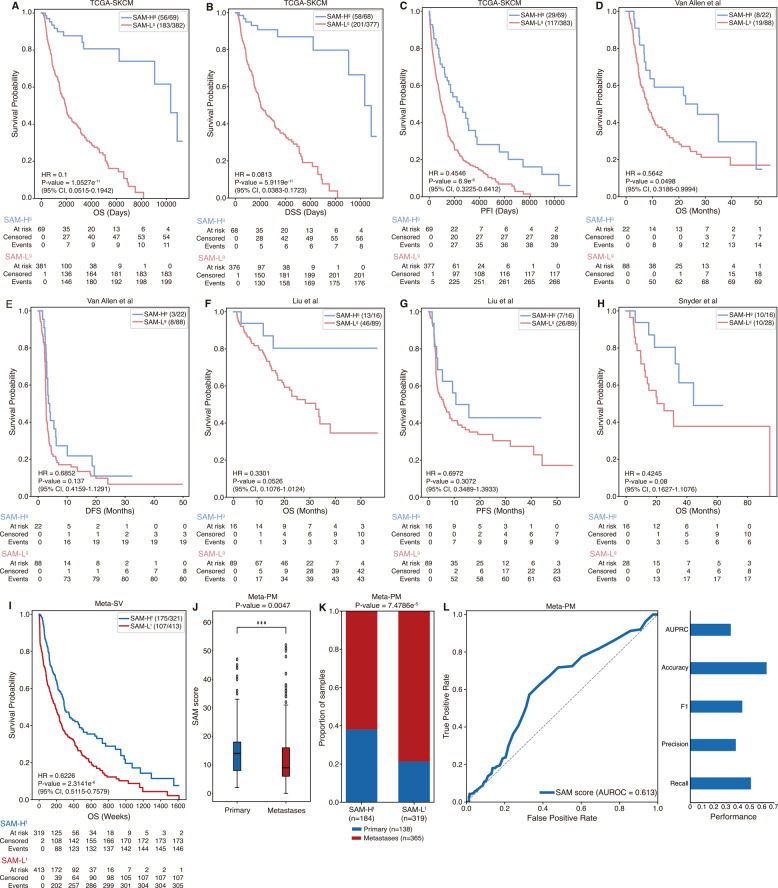
Validation for SAM pairs based on TCGA, cBioportal, and GEO datasets. **A-H** The survival analysis results with Kaplan–Meier plots for two groups (SAM-H^g^ and SAM-L^g^) from melanoma somatic mutation datasets, which are **A-C** TCGA-SKCM, **D**, **E** Van Allen et al., **F**, **G** Liu et al., and **H** Snyder et al. **I** The survival analysis result with Kaplan–Meier plot for two groups (SAM-H^t^ and SAM-L^t^) from Meta-SV. **J** Box plot showing the differences in SAM scores between primary and metastases tumor samples in Meta-PM. **K**, **L** Prediction performance using the SAM score to Meta-PM. Statistical significance was measured by two-sided Student’s *t*-test (***P-value < 0.001), two-sided Fisher’s exact test, and Cox proportional hazards regression analysis. The denominator is the number of all patients for each group, and the numerator is the number of patients for each group who are alive from all Kaplan-Meier plots

To investigate whether the number of SAM pairs with both genes impaired was associated with improved prognosis in patients, we conducted survival validation using somatic mutation and clinical datasets from TCGA-SKCM. This analysis is based on the hypothesis that patients with a high number of co-impaired SAM pairs would have better survival outcomes than those with fewer. Therefore, we calculated the SAM score for each melanoma patient and stratified them into two groups (high/low-score group) to assess the relationship between SAM burden and survival (see “Methods”). We found that the high-score groups (SAM-H^g^) showed significantly better overall survival (OS), disease-specific survival (DSS), and progression-free survival (PFI) outcomes than the low-score group (SAM-L^g^) in TCGA-SKCM (Fig. 2A-C; *P*-value < 0.05 was considered significant). These results suggest that the number of SAM pairs with both genes impaired could be a useful biomarker for predicting survival outcomes in melanoma patients.

### External validation using the genomic datasets from cBioPortal

We conducted external validations using genomic and clinical datasets to assess whether the SAM-based approach observed in the TCGA could be reproduced in independent datasets. We used three somatic mutation and clinical datasets: Van Allen et al., Liu et al., and Snyder et al. from cBioPortal (Supplementary Table 1; see "Methods") [25–27]. We performed variant annotation using ANNOVAR to derive the SAM score after calculating the GVB score, which follows the same procedure as used in the TCGA analysis. Then, we stratified patients into two groups (high/low-score group) based on the SAM score to evaluate survival differences in independent datasets. Among the 325 SAM pairs identified from the TCGA-SKCM, 167 pairs in Van Allen et al., 225 pairs in Liu et al., and 110 pairs in Snyder et al. could stratify patients into two groups for each dataset. We found that the SAM-H^g^ group showed a trend toward better survival outcomes compared to those in the SAM-L^g^ group across all datasets (Fig. 2D-H; *P*-value < 0.05 was considered significant). These results suggest the generalizability of the number of SAM pairs with both genes impaired as potential survival prediction biomarkers in independent genomic datasets.

### External validation using the transcriptomic datasets from GEO

Next, we conducted external validation to assess whether the SAM pairs could predict patient survival by using transcriptomic datasets. To validate the SAM pairs with similar approaches conducted in validation using genomic datasets, we created a meta-cohort, Meta-SV (SurVival), by integrating five independent datasets: GSE22153, GSE22154, GSE54467, GSE59455, and TCGA-SKCM (Supplementary Table 2; Supplementary Data S4; see “Methods”). We found that the SAM-H^t^ group had significantly better survival outcomes than the SAM-L^t^ group (Fig. 2I; HR = 0.6226; *P*-value = 2.3414e^-6^) using 140 pairs identified in Meta-SV. This result suggests that the number of SAM pairs with both genes inactivated may serve as a prognostic indicator of patient survival, even in an independent transcriptomic dataset.

We also conducted additional external validation by using different transcriptomic datasets to assess whether SAM pairs could differentiate between primary and metastases tumor samples. To ensure the differences, we constructed a meta-cohort, Meta-PM (Primary and Metastases), by integrating five independent datasets: GSE7553, GSE8401, GSE15605, GSE46517, and GSE65904 (Supplementary Table 3; Supplementary Data S5; see “Methods”). We compared SAM scores between primary and metastases tumor samples using 115 pairs identified in Meta-PM. We found that primary tumors had significantly higher SAM scores than metastases tumors (Fig. 2J; two-sided Student’s *t*-test; *P*-value = 0.0047). We also found that the SAM scores were accurately predictive for tumor sample types (Fig. 2K, L; two-sided Fisher’s exact test; *P*-value = 7.4786e^-5^; AUROC = 0.613). These results suggest that the number of SAM pairs with both genes inactivated may reflect transcriptional differences associated with metastatic status.

### Immune cell infiltration and single-cell RNA sequencing analysis with SAM score stratification

Metastatic potential could emerge from interactions between tumor cells and the surrounding immune-stromal niche [28–30]. Therefore, we further evaluated whether SAM groups show the corresponding difference in immune infiltration and single-cell tumor states across meta-cohorts and single-cell RNA sequencing (scRNA-seq) datasets.

**Fig. 3:**
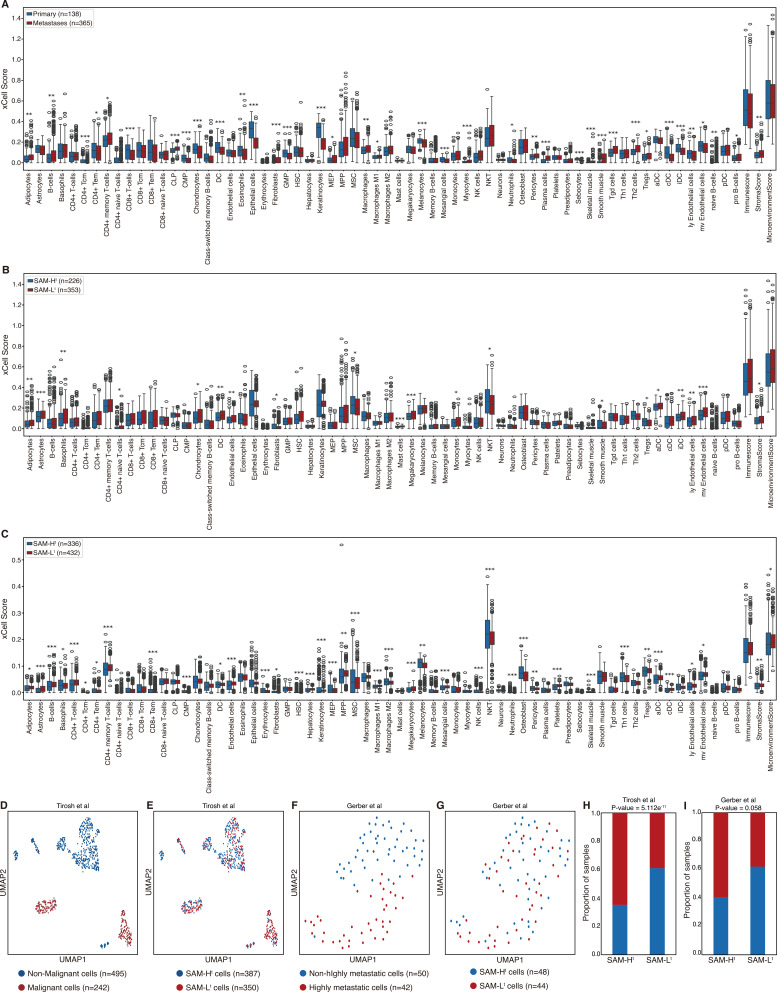
Identification of immune context and single-cell concordance using SAM stratification. **A-C** Comparison of immune cell infiltration between two groups: **A** primary and metastases tumor samples in Meta-PM, **B** SAM-H^t^ and SAM-L^t^ in Meta-PM, and **C** SAM-H^t^ and SAM-L^t^ in, Meta-SV. **D**, **E** UMAP visualization of 737 cells from Tirosh et al. grouped by **D** malignant/non-malignant cells, and **E** SAM-H^t^/SAM-L^t^ cells. **F**, **G** UMAP visualization of 92 cells from Gerber et al. grouped by **F** highly metastatic/non-highly metastatic cells, and **G** SAM-H^t^/SAM-L^t^ cells. **H**, **I** Prediction performance of stratification with SAM score to **H** Tirosh et al., and **I** Gerber et al. Statistical significance was measured by two-sided Student’s *t*-test (*P-value < 0.05, **P-value < 0.01, and ***P-value < 0.001), and two-sided Fisher’s exact test. Boxplot shows the median value, outliers, and interquartile range (IQR) as bounds of the box with whiskers extending to the upper and lower quartiles ± 1.5 times the IQR

We investigated whether there were relationships between groups classified by SAM score and tumor-infiltrating immune cells using xCell (Fig. 3A-C; two-sided Student’s t-test; *P*-value < 0.05 was considered significant; see “Methods”) [31]. We found that metastases and SAM-L^t^ groups from Meta-PM and SAM-L^t^ groups from Meta-SV have significantly high enrichment scores for stroma score and adipocytes. We also found that metastases and SAM-L^t^ groups from Meta-PM and SAM-L^t^ groups from Meta-SV have a high enrichment score trend for B-cells, CD4+ memory T-cells, CD4+ T-cells, hepatocytes, macrophages, macrophages M1, mast cells, microenvironment score, monocytes, and MPP. Conversely, we found that primary and SAM-H^t^ groups from Meta-PM and SAM-H^t^ groups from Meta-SV have a high enrichment score trend for CMP, eosinophils, MSC, neurons, neutrophils, and sebocytes. 

We also investigated the biological relevance between cell type and SAM score-based classification using two melanoma scRNA-seq datasets, Tirosh et al. and Gerber et al (Supplementary Data S6, S7; see “Methods”) [32, 33]. Tirosh et al. previously mapped 737 cells from GSE72056 into B cell, CAF, endothelial cell, macrophage, malignant, NK cell, T cell CD4, and T cell CD8. Therefore, we additionally classified 737 cells into two groups: malignant and non-malignant (Fig. 3D; Supplementary Fig. 2). Gerber et al. previously mapped 92 cells from GSE81383 into three groups: highly metastatic tumors (group 1), high-grade primary tumors (group 2), and invasive primary tumors (group 3), using a self-organizing map (Supplementary Fig. 2). Also, Gerber et al. discovered that group 1 was anti-correlated with groups 2 and 3. Therefore, we additionally classified 92 cells into two groups: highly metastatic (group 1) and non-highly metastatic (group 2 and 3) (Fig. 3F). We found that malignant cells were significantly enriched in SAM-L^t^ while non-malignant cells were significantly enriched in SAM-H^t^ from Tirosh et al. (Fig. 3E, H; two-sided Fisher’s exact test; *P*-value = 5.112e^-11^). We also found that the highly metastatic cells were enriched in SAM-L^t^ while non-highly metastatic cells were enriched in SAM-H^t^ from Gerber et al. (Fig. 3G, I; two-sided Fisher’s exact test; *P*-value = 0.058). Moreover, we found that there were associations between the SAM groups and groups 1 to 3 annotated by Gerber et al (Supplementary Table 4; Chi-square test; *P*-value = 0.115). For cell type information, we found that there were significant associations between the SAM groups and cell types in Tirosh et al. (Supplementary Table 5; Chi-square test; *P*-value = 1.551e^-11^). In Gerber et al., we found that there were associations between SAM groups and cell types (Supplementary Fig. 2; Supplementary Table 6; Chi-square test; *P*-value = 0.329). These results suggest that the SAM score-based stratification can robustly distinguish malignant cells from non-malignant cells and metastatic cells from non-metastatic cells at single-cell resolution, consistent with previously established tumor state annotations.

### Identification of SAM-associated pathways through functional enrichment analysis

We confirmed that SAM score-based classification could reliably capture metastatic potential and malignant cell state from meta-cohort and single-cell datasets.

**Fig. 4:**
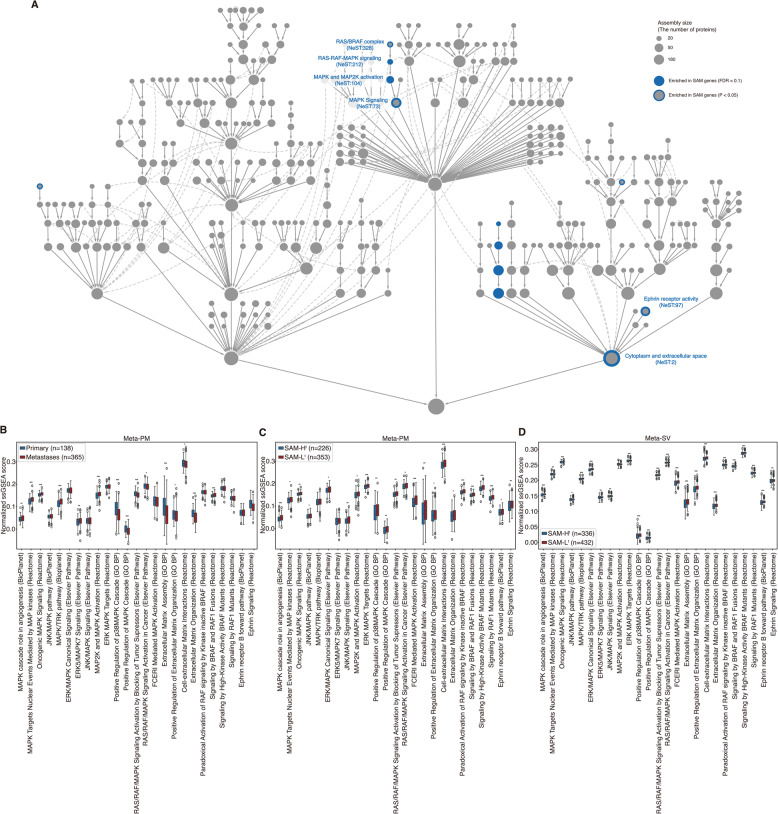
Functional enrichment analysis and evaluation of pathways. **A** Protein assemblies related to SAM pairs are represented on the NeST. **B-D** Boxplot showing the differences in NES from ssGSEA between two groups: **B** primary and metastases tumor samples in Meta-PM, **C** SAM-H^t^ and SAM-L^t^ in Meta-PM, and **D** SAM-H^t^ and SAM-L^t^ in Meta-SV. Statistical significance was measured by the hypergeometric test and two-sided Student’s *t*-test (*P-value < 0.05, **P-value < 0.01, and ***P-value < 0.001). Boxplot shows the median value, outliers, and interquartile range (IQR) as bounds of the box with whiskers extending to the upper and lower quartiles ± 1.5 times the IQR

We investigated whether genes in SAM pairs are associated with melanoma metastasis in terms of functional roles. Therefore, we performed functional enrichment analysis on the genes in SAM pairs using NeST protein assemblies with GSEApy (see “Methods”) [34, 35]. We found that 12 protein assemblies, including the MAPK and MAP2K activation, RAS-RAF-MAPK signaling, MAPK signaling, RAS/BRAF complex, and ephrin receptor activity were enriched in genes in SAM pairs (Fig. 4A; Supplementary Data S8; hypergeometric test; FDR < 0.1 was considered significant). We further investigated whether those assemblies were associated with melanoma metastasis from previous studies. MAPK has been known to promote metastasis in melanoma [36]. Also, RAS-RAF-MAPK signaling has been known to drive melanoma invasion and metastatic progression by inducing epithelial-mesenchymal transition (EMT) [37, 38]. Moreover, ephrin receptors could contribute to melanoma progression and metastasis by regulating cell migration and promoting angiogenesis [39–41]. These results suggest that SAM pairs are functionally associated with metastasis-promoting pathways, supporting their role in melanoma progression.

Given that the MAPK-, ephrin-, and cytoplasm/extracellular space-related assemblies were associated with genes in SAM pairs, we also investigated the activity of individual pathways associated with MAPK, ephrin and cytoplasm/extracellular space across two meta-cohorts using single-sample gene set enrichment analysis (ssGSEA) from GSEApy (see “Methods”). We found that in the Meta-PM, the metastases samples have significantly higher normalized enrichment scores (NES) than primary samples for most of the pathways(Fig.4B; two-sided Student’s *t*-test; *P*-value < 0.05 was considered significant). Consistently, we also found the trend that SAM-L^t^ samples have elevated NES values relative to SAM-H^t^ samples across both meta-cohorts (Fig. 4B-D; two-sided Student’s *t*-test; *P*-value < 0.05 was considered significant). These results suggest that the SAM-L^t^ group retains the high MAPK- and ephrin-related signaling patterns typical of the metastases tumors, whereas the SAM-H^t^ and primary group are characterized by attenuated metastasis-promoting signaling.

### Prediction of primary and metastases samples in transcriptome data using ML

**Fig. 5:**
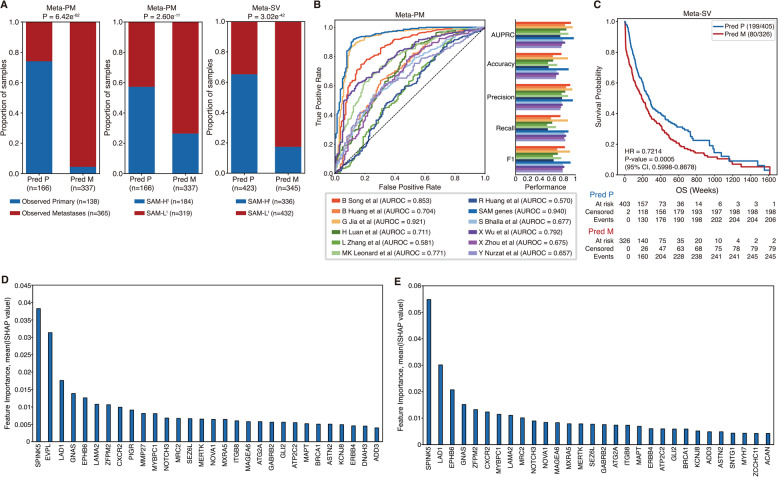
Predict primary and metastases samples using ML. **A** Prediction performance using ML constructed by expression profiles of genes in SAM pairs. **B** ML performances for each metastatic melanoma-associated biomarker from previous research and genes in SAM pairs of within-study prediction of Meta-PM. **C** The survival analysis result with Kaplan-Meier plot between predicted primary and metastases from across-study prediction for Meta-SV. The denominator is the number of all patients for each group, and the numerator is the number of patients for each group who are alive. **D-E** The top 30 features according to the mean (|SHAP value|) of genes in SAM pairs from the **D** within-study prediction and **E** across-study prediction, trained by Meta-PM. Statistical significance was measured by two-sided Fisher’s exact test and Cox proportional hazards regression analysis

Given that the co-impaired or -inactivated SAM pair burden could predict melanoma patients' prognosis and stratify the tumor sample types, we further investigated whether genes in SAM pairs could classify primary and metastases tumor samples and reveal the major genetic determinants of this distinction. Therefore, we constructed an SAM-based ML model to determine whether genes in SAM pairs could distinguish metastases from primary tumor samples in melanoma (see “Methods”). To develop an SAM-based model, we found that a balanced random forest (BRF) classifier could predict tumor samples accurately and robustly compared to other ML methods (Supplementary Fig. 3A; two-sided Student's *t*-test; *P*-value < 0.05 was considered significant). Next, we performed a within-study prediction trained by selected expression signatures of 138 genes in SAM pairs from the Meta-PM using a BRF classifier with leave-one-out cross-validation (LOOCV). Then, we compared the performance of the SAM-based model to the other performances of 11 ML models. The 11 ML models were established in the same approach as the SAM-based model but trained by different selected expression signatures of melanoma-associated genes reported from 11 previous studies (Supplementary Data S9) [11, 42–51]. We found that the SAM-based model made accurate predictions for sample types and SAM groups (Fig. 5A; two-sided Fisher's exact test; *P*-value < 0.05 was considered significant). We also found that the SAM-based model outperformed in predicting tumor sample types compared to the other 11 models (Fig. 5B; AUROC = 0.940; Supplementary Fig. 3B; two-sided Student's *t*-test; Supplementary Data S10). These results suggest that genes in SAM pairs could be better biomarkers to predict tumor sample types compared to other known melanoma-metastasis associated biomarkers.

We further investigated whether predicted primary samples have improved survival outcomes than predicted metastases samples from across-study prediction (see "Methods"). We constructed an across-study prediction model by training Meta-PM with a BRF classifier to predict primary and metastases samples from Meta-SV. We measured the performance for predicting the patient’s survival using Cox proportional hazards regression analysis. We found that the balanced random forest classifier accurately predicted patients’ survival outcome compared to other ML methods (Supplementary Table 7). We also found that predicted primary samples have significantly better survival outcomes than predicted metastases samples in Meta-SV (Fig. 5C; HR = 0.7214; *P*-value = 0.0005). Moreover, we found that the SAM-based model consistently outperformed most of the reference 11 models in predicting prognosis (Supplementary Table 8). These results suggest that genes in SAM pairs could also be better biomarkers compared to other melanoma metastasis-related biomarkers when predicting patients’ survival prognosis.

Finally, to interpret SAM-based model behavior, we used SHAP analysis to identify high-impact genetic features in within-study prediction and across-study predictions (see “Methods”) [52]. Among the common 106 genes used as features in the ML model for within- and across-study predictions, we found that 26 genes were the common top 30 genes that were highly important features for each prediction model (Fig. 5D-E; Supplementary Data S11). This result suggests that those genes shared across both models supported their robustness as predictive biomarkers for melanoma metastasis.

### Discovery of potential anti-metastasis drugs for melanoma

**Fig. 6:**
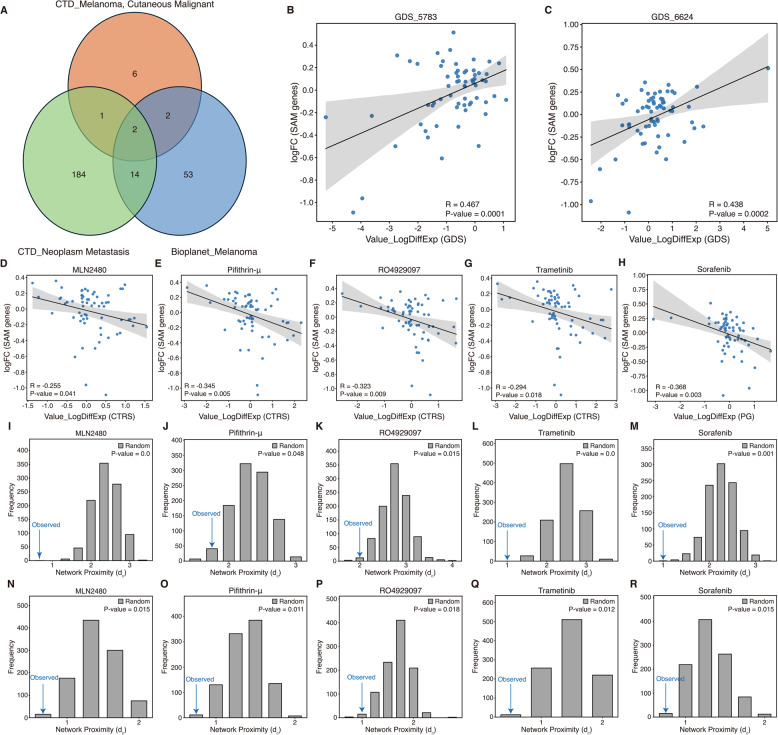
Identifying candidate anti-metastasis drugs for melanoma. **A** Identification of genes associated with both melanoma and metastasis in the PPI network from 2 public databases. **B**,** C** Gene expression correlation between iLINCS disease-related signatures from and significant signatures from Meta-PM restricted to genes in SAM pairs. **D-H** Gene expression correlation between compounds’ signatures, which were **D** MLN2480, **E** Pifithrin-µ, **F** RO4929097, **G** Trametinib, and **H** Sorafenib, and significant signatures from Meta-PM restricted to genes in SAM pairs. **I-M** Network proximity between compounds’ target genes, which were **I** MLN2480, **J** Pifithrin-µ, **K** RO4929097, **L** Trametinib, and **M** Sorafenib, and genes associated with both melanoma and metastasis in the PPI network. **N-R** Network proximity between compounds’ target genes, which were **N** MLN2480, **O** Pifithrin-µ, **P** RO4929097, **Q** Trametinib, and **R** Sorafenib, and genes in SAM pairs in the PPI network. Statistical significance of the correlation was measured by Pearson correlation, and the network proximity was measured by empirical distribution

We observed that expression signatures of genes in SAM pairs could accurately and robustly predict not only sample types but also melanoma patients’ prognosis in both within- and across-study predictions. Therefore, we investigated their potential utility to discover candidate drugs that could inhibit melanoma metastasis. Before we discovered candidate anti-metastasis drugs for melanoma, we assessed whether the significant expression signatures of genes in SAM pairs from Meta-PM could be similar to those in public-domain transcriptomic datasets (see “Methods”). We performed differential gene expression (DGE) analysis on the Meta-PM to submit the expression profiles of 66 DEGs in SAM pairs into iLINCS (Supplementary Data S11; FDR < 0.1 was considered significant) [24]. We found that there were significant positive correlations between Meta-PM and public-domain transcriptional datasets that used control for normal and test for malignant melanoma or metastatic-growth phase melanoma (Fig. 6B, C; Pearson correlation; *P*-value < 0.05 was considered significant). These results suggest that our expression signatures are representative and suitable for candidate anti-metastasis drug screening for melanoma.

To identify candidate anti-metastasis drugs for melanoma, we hypothesized that compounds with expression profiles of DEGs reverse to those observed in metastatic melanoma could serve as candidate anti-metastatic drugs. The hypothesis is based on compounds that could be used as a therapeutic option for disease if compounds reverse disease-associated gene expression profiles [53–55]. We found that 16 compounds, including MLN2480, pifithrin-µ, RO4929097, and trametinib, showed significantly reverse expression profiles of DEGs in SAM pairs between Meta-PM and the cancer therapeutics response signatures library from iLINCS (Fig. 6D-G; Supplementary Table 9; Pearson correlation; *P*-value < 0.05 was considered significant). We also found that 6 compounds, including sorafenib, showed significant reverse expression profiles of DEGs in SAM pairs between Meta-PM and pharmacogenomics transcriptional signatures library from iLINCS (Fig. 6H; Supplementary Table 10; Supplementary Data S12; Pearson correlation; *P*-value < 0.05 was considered significant). These results suggest that 22 compounds would be candidate anti-metastasis drugs for melanoma in terms of drug repurposing based on gene expression signatures. Network biology has offered an important perspective on the relationship between drug and disease. Proteins related to similar phenotypes tend not to exist randomly scattered but interact and exist closely with each other in the interactome [56, 57]. Also, a drug’s efficacy could be inferred from drug-disease proximity in the interactome [58]. Therefore, we hypothesized that in the PPI network, 229 genes from 325 SAM pairs could form a single module and be proximal to genes commonly associated with malignant melanoma and neoplasm metastasis. For network analyses, we constructed a PPI network consisting of 15,867 nodes and 235,783 edges for a largest connected component (LCC) from the STRING database (see “Methods”) [59]. We also used common genes of melanoma and metastasis from BioPlanet and the CTD database (Fig. 6A) [60]. We found that genes in SAM pairs form a module and the module was proximal to common genes associated with both malignant melanoma and neoplasm metastasis in the PPI network (Supplementary Fig. 4; *P*-value < 0.05 was considered significant; see "Methods"). These results suggest that SAM pairs could be associated with an anti-metastasis effect in terms of network biology. We also hypothesized that candidate anti-metastasis drugs for melanoma could be proximal to genes associated with both malignant melanoma and neoplasm metastasis or genes in SAM pairs (see "Methods"). To calculate network proximity using drug target genes, we selected candidate compounds that have at least one target gene from the Cancer Therapeutics Response Portal (CTRP) database [61]. We found that 12 compounds, including MLN2480, pifithrin-µ, RO4929097, sorafenib, and trametinib, were significantly proximal to genes commonly associated with both malignant melanoma and neoplasm metastasis (Fig. 6I-M; Supplementary Table 11; *P*-value < 0.05 was considered significant). Moreover, we found that 5 compounds, MLN2480, pifithrin-µ, RO4929097, sorafenib, and trametinib, were significantly proximal to genes in SAM pairs (Fig. 6N-R; Supplementary Table 12; *P*-value < 0.05 was considered significant). These results suggest that MLN2480, pifithrin-µ, RO4929097, sorafenib, and trametinib could be candidate anti-metastasis drugs for melanoma in terms of drug repurposing based on network biology.

Finally, we investigated whether the candidate drugs had metastasis-suppressive effects from previous studies. Trametinib, a MEK inhibitor for metastatic melanoma, could hinder tumor metastasis in a mucosal melanoma metastatic model used with sapanisertib as a combination therapy [62, 63]. Gampa et al. demonstrated that panRAF inhibitor MLN2480 could represent a candidate for treating melanoma brain metastasis with its enhanced brain delivery profile [64]. Also, Jayaprakash et al. showed that notch signaling inhibition with RO4929097 could be a promising therapeutic strategy to improve the efficacy of radiation therapy in melanoma in terms of interrupting the development of metastasis [65]. Moreover, Takeda et al. observed that sorafenib could decrease cell migration and invasion in vitro and inhibit metastasis in vivo with c-Kit aberration [66]. However, previous research didn’t demonstrate that pifithrin-µ has a therapeutic effect on metastatic skin cutaneous melanoma but could have an effect on other cancer type, for example NSCLC [67]. These results suggest that MLN2480, RO4929097, sorafenib, and trametinib would be candidate anti-metastasis drugs for melanoma in terms of the literature review.

## Discussion

In this study, we have demonstrated that the novel pairwise biomarker, SAM, might be associated with anti-metastasis in melanoma. While SL has proven valuable in precision oncology, its utility in metastasis prediction or suppression remains limited. This is largely due to the co-impairment of the biological background of SL, of genes typically resulting in cell death, making it difficult to evaluate whether invasion or migration was inhibited before apoptosis [16–18, 68] Therefore, we conceptualized the SAM interaction, which potentially improves melanoma patients’ prognosis by suppressing metastasis without necessarily inducing cancer cell death when both genes are impaired. Based on this concept, we could systemically identify SAM pairs from a large-scale melanoma cohort by excluding known SL pairs and those containing at least one essential gene.

We demonstrated the robustness and clinical relevance of SAM pairs across multiple datasets and approaches. Although the external genomic datasets were smaller than TCGA, we could observe consistent survival trends and significant differences between primary and metastases tumors using SAM scores. We also demonstrated that the SAM-based ML models’ predictive performance was superior to existing melanoma metastasis-related biomarkers from within- and across-study predictions. Notably, several previous studies identified melanoma metastasis-associated biomarkers by directly comparing primary and metastases tumor profiles. In contrast, we identified SAM pairs without pre-stratifying by metastatic status in melanoma based on clinical stage or molecular subtype, further underscoring their potential generalizability. 

We also explored the interpretability of SAM pairs using immune cell infiltration analysis, scRNA-seq analysis, functional enrichment analysis, and feature importance analysis from ML models. We found that metastases and SAM-L^t^ samples have higher stroma and microenvironment scores compared to primary and SAM-H^t^. The melanoma cell invasion could be facilitated by the dynamic interaction between melanoma and stroma that results in melanoma metastasis [69]. Furthermore, cells and factors from the tumor microenvironment could play a vital role in preparing the premetastatic niche to promote metastasis formation [70]. We also found that certain cell types were enriched in SAM-H^t^ samples, and these same cell types have previously been associated with cancer metastasis, including melanoma (Supplementary Fig. 2; Supplementary Tables 5, 6). For example, endothelial cells could promote tumor metastasis by enhancing tumor angiogenesis with expressing Bcl-2 [71]. In contrast, previous studies have reported that NK cells could suppress melanoma metastasis [72, 73]. Moreover, we found that several SAM-related pathways, including the MAPK, RAS-RAF-MAPK, and ephrin receptor signaling, were linked to metastatic progression. At last, we found that some genes contributing to the ML models’ high predictive performance have prior associations with metastasis (Fig. 5D, E). For example, the miR500-SPINK5/STAT3 axis could promote melanoma cell metastasis [74]. Also, significant reduction of EPHB6 gene expression could be associated with melanoma progression to metastatic melanoma [39, 75]. Moreover, ZFPM2-AS2 could facilitate cell proliferation and migration in cutaneous malignant melanoma through miR-650/NOTCH1 signaling [76].

However, there are several limitations in this study. First, there were differences in clinical information of patients, such as demographics and treatment history, between datasets. Some somatic variants might differ by race, gender, and treatment history. Also, melanoma patients’ survival prognosis would be affected by stage and treatment history. Therefore, those differences would affect the identification and validation of SAM pairs, leading to a limitation in the applicability to other patient groups. Second, external datasets that have incomplete or lacking information might affect the survival analysis performed during the validation process. The datasets from cBioPortal and GEO used in external validation were limited in size or information compared to TCGA. For example, important clinical variables such as treatment history, cancer stage, and survival time were missing or incomplete. Therefore, accurate survival analysis in external datasets may be hindered, resulting in inconsistencies between internal and external validation results. At last, all the analyses conducted in this study have not been completely experimentally validated. This study relied solely on in silico, data-driven analyses to discover and validate SAM pairs and candidate anti-metastatic drugs in melanoma. Therefore, further experiments will be required to ensure the reliability

## Conclusion

In summary, this study provides a comprehensive framework to identify SAM interactions as metastasis-predictive biomarkers and potential leads for anti-metastatic drug discovery in melanoma from multi-omics and -cohorts data. Therefore, this study may inspire other researchers by improving the clinical applicability of pairwise biomarkers and potentially introducing a new paradigm in the treatment of metastatic melanoma.

## Methods

### Discovery of primary SAM pairs from the TCGA dataset

To identify SAM pairs, we used somatic mutation and clinical data from the TCGA skin cutaneous melanoma (SKCM) cohort. Specifically, we downloaded the somatic mutation data file (mc3.v.0.2.8.PUBLIC.maf) and the corresponding clinical outcome data file (TCGA-CDR-SupplementalTableS1.xlsx) from the Pan-cancer Atlas (PanCanAtlas) via the NIH Genomic Data Commons (GDC) [21, 22].

We selected patients’ samples from the TCGA CDR, which contained clinical information on gender, race, age at initial pathologic diagnosis, vital status, and overall survival time (OS). Using these samples, we calculated gene-wise variant burden (GVB), previously described by Park et al., to quantify functional damage to individual gene [23]. We calculated GVB scores based on loss-of-function (LOF) mutations and missense mutations filtered by SIFT scores. All mutations passed the filter in the genome data. We considered nonsense mutation, frameshift indel, and splice site mutation as LOF mutations [77]. Missense mutations with a SIFT score of 0 were replaced with 10^− 8^ for computational stability. The GVB scoring method was implemented as follows:

1 $$\:G_i=\left\{v\:\right|\:v\:with\:a\:SIFT\:score\:less\:than\:0.7\}$$

2 $$\:{{}_{adj}v}_{j}=\left\{\begin{array}{c}SIFT\:score,\:\:\:if\:{v}_{j}\:\:and\:heterozygote\\\:\\\:{\left(SIFT\:score\right)}^{2},\:\:\:if\:{v}_{j}\:\:and\:homozygote\:\end{array}\right.$$

3 $$\:GVB\left(G_i\right)=\left\{\begin{array}{c}1,\:\:if\:n\left(G_i\right)=0\\\:\\\left(\prod_{j=1}^{n}{{}_{adj}v}_{j}\right)^{1/n},\:\:if\:n\left(G_i\right)>\:0\:\end{array}\right.\:$$

To calculate the GVB score, we only considered the variants *v* with a SIFT score less than 0.7 as potentially deleterious for each *ith* gene *G*_*i*_. We also calculated the adjusted SIFT score for each variant, _*adj*_*v*, according to genotype, as homozygous variants considered more damaging than those in a heterozygote. The GVB score for each gene *G*_*i*_ with *n* deleterious variants was calculated as the geometric mean of _*adj*_*v*. If the gene *G*_*i*_ has at least one LOF mutation, we considered GVB(*G*_*i*_) as 10^-8^.

We defined genes with GVB < 0.2 as impaired using variant summary data and pLoF data metrics by gene (Supplementary Fig. 1; Supplementary Data S1; see “Methods”) [78, 79]. Using the threshold, we categorized patients into four groups based on the impairment status of two genes in a candidate pair: (i) Patients with both GVBs < 0.2 (patients with both genes damaged), (ii) Patients with only one gene with a GVB < 0.2 (patients with only one gene damaged), (iii) Patients with only the other gene with a GVB < 0.2 (patients with only the other gene damaged), (iv) Patients with both GVBs ≥ 0.2 (patients with both genes were not damaged).

We used the Cox proportional hazards regression analysis with group (iv) as the reference group to evaluate prognostic significance. The Cox proportional hazards regression model included covariates for gene pair group, gender, race, and age at initial pathologic diagnosis. The hazard ratio (HR) was measured using CoxPHFitter from the Python lifelines package with a penalizer value of 0.5 to avoid convergence issues [80]. We considered candidate SAM pairs if the following conditions were met: Patient group (i) has a statistically significant better survival outcome than the patient group (iv) (HR < 1, *P*-value < 0.05). However, the patient groups (ii) and (iii) should have worse outcomes (HR > 1) or non-significant outcomes (*P*-value ≥ 0.05) than the patient group (iv).

### Statistical determination to identify the gene-level impairment threshold

To identify robustly impaired genes from the GVB score matrix *M*_*g,i*_, (rows for gene from gene set *U* and columns for patients from TCGA-SKCM), we employed an expected value (EV) controlled thresholding framework. For each gene *g* and threshold *T*, the impairment frequency across melanoma samples is defined as below:

4 $$\:{x}_{g}\left(T\right)=\sum_{i=1}^{n}\:1\:\{{M}_{g,i}\:<\:T\}\:$$

The impaired gene set was constructed at varying thresholds and *k*, which is the minimal patient counts defined in the formula below:

5 $$\:I(T,k)=\:\left\{{x}_{g}\right(T)\:\ge\:\:k\:\}\:$$

The expected false inclusion rate was modeled by a binomial or beta-binomial distribution, depending on the estimated over-dispersion parameter *ρ* with emperical mean *µ* and variance of *x*_*g*_(*T*):

6 $$\mu\ =\ \frac{\bar{x}}{n}, \rho\ =\ \frac{Var(x)/(n\mu\ (1\ -\ \mu\ ))\ -\ 1}{n\ -\ 1}$$

7 $$\mathit\:\textit{EV}\mathit{\left(k\right)}=\:\left|U\right|\bullet\:\:P(X\geq\:\:k)\:$$

If *ρ* > 0, *x*_*g*_(*T*) follows a beta-binomial distribution:

8 $$\alpha\;=\;\mu(\frac1\rho\;-\;1),\beta=(1-\;\mu)(\frac1\rho\;-\;1)$$

Otherwise, we used a simple binomial model. We selected the smallest k^*^(*T*) when satisfying *EV(k)* ≤ *EV*_*target*_ (0.5 or 1.0). We also estimated over-dispersion adjusted parameters (*α*, *β*) from the empirical mean and variance of *x*_*g*_(*T*). This EV-controlled approach contains the number of genes expected to appear impaired by change at any threshold.

 To ensure the reproducibility of the GVB threshold, we quantified stability with median Jaccard similarity between impaired gene sets from 1,000 random half-splits of the cohort recomputing *I*_*L*_ and *I*_*R*_ with scaled *k*:

9 $$stab(T)\ =\ median\left(\frac{|I_L\ \cap\ I_R|}{|I_L\ \cup\ I_R|}\right)$$

For each *T*, range from 0.1 to 0.9 in increments of 0.1, we also computed biological enrichments of *I*(*T*, k^*^) against three orthogonal reference sets: (i) functionally constrained genes (LOEUF ≤ 0.35; gnomAD v2.1.1), (ii) LOF-tolerant genes (LOEUF ≥ 1.0), and (iii) skin cutaneous melanoma-related somatic oncogenic genes (ClinVar). Significance was tested by Fisher’s exact test (Haldane-Anscombe correction and FDR-BH adjustment). Candidate thresholds were retained if they met the criteria (i) stability ≥ 0.6 and (ii) set-size constraints (50 ≤ |*I*| ≤ 0.5⋅|*U*|). The final threshold T^*^ was defined as the center of the largest contiguous region that met these criteria.

### Identification of precise SAM pairs from candidate SAM pairs

We applied the two sequential criteria to identify high-confidence SAM pairs from preliminary candidate pairs. We defined that candidate pairs were retained only if they met all the following conditions: The gene pair should (i) not be an SL gene pair and (ii) not contain at least one essential gene, but only for non-essential genes (Fig. 1B).

Therefore, (i) we excluded the gene pairs listed in two SL resources, which were experimentally or computationally discovered: 6,033 SL gene pairs provided by Lee et al. and 37,536 human SL gene pairs provided by SynLethDB (v3.0) [18, 81]. Moreover, (ii) we used 19,062 gene essentiality profiles provided by Nichols et al [82]. We refined the pairs that have only non-essential genes from Nichols et al.

### SAM score

We defined the SAM score to quantify the number of SAM pairs in which both genes are either genetically impaired or transcriptionally inactivated. We used the SAM score to compare primary versus metastases tumor profiles and evaluate survival prognosis.

In the genomic dataset, we defined whether a gene was damaged by using the GVB score. In the transcriptomic dataset, we defined gene inactivation when it was below the 1/3-quantile across samples [16, 18]. The SAM score for each patient was calculated by summing the number of SAM pairs in which both genes were impaired or inactivated:

10 $$\:SAM\:score=\sum\:_{n=1}^{N}S\left(n\right)\:$$

11 $$\:S\left(n\right)=\left\{\begin{array}{c}0,\:\:\:if\:both\:genes\:in\:the\:nth\:SAM\:pair\:are\:not\:impaired\:\left(inactivated\right)\\\:\\\:1,\:if\:both\:genes\:in\:the\:nth\:SAM\:pair\:are\:impaired\:\left(inactivated\right)\end{array}\right.$$

This metric is based on the hypothesis that a higher number of co-impaired or -inactivated SAM pairs is associated with improved patient prognosis by suppressing melanoma metastasis. Therefore, we stratified patients into high (SAM-H^g^ in the genomic and SAM-H^t^ in the transcriptomic dataset) and low (SAM-L^g^ in the genomic and SAM-L^t^ in the transcriptomic dataset) SAM score groups using the mean SAM score as the threshold within each dataset.

### Internal validation of SAM pairs in the TCGA dataset

To assess the prognostic value of SAM pairs, we conducted internal validation using somatic data from the TCGA-SKCM. Specifically, we tested whether patients with a higher number of SAM pairs that have both co-impaired genes had improved survival outcomes compared to those with fewer such pairs. Therefore, we performed survival analysis with the Cox proportional hazards regression using the same clinical covariates used to discover preliminary SAM pairs and TMB as an additional covariate. We calculated TMB by using the following formula from Wang et al [83].

12 $$\:{TMB}_{patient}\:=\:{T}_{patient}\times2.0\:+\:{NT}_{patient}\:\times\:1.0\:$$

From the formula, *T*_*patient*_ is the total number of truncating mutations, and the *NT*_*patient*_ is the total number of non-truncating mutations. Wang et al. considered that nonsense, frameshift indel, and splice site mutations as a truncating mutation. Also, Wang et al. considered missense, in-frame indel, and nonstop mutations as non-truncating mutations. We used a likelihood ratio test (LRT) to determine whether TMB could be a useful predictor in the Cox proportional hazards regression model (Supplementary Data S3).

We evaluated three clinical outcome endpoints in internal validation: overall survival (OS), disease-specific survival (DSS), and progression-free survival (PFS). We stratified patients into SAM-High (SAM-H^g^) and SAM-Low (SAM-L^g^). We used the SAM-L^g^ group as the reference group by setting the panelizer value to 0.01 to ensure model convergence.

### Preparation and preprocessing of datasets for external validation

To validate the utility of SAM pairs in predicting melanoma prognosis and distinguishing between primary and metastases tumors, we curated and preprocessed external datasets, including genomic and transcriptomic profiles.

We used three independent melanoma cohorts with somatic mutation and clinical data from cBioPortal, which were Liu et al., Snyder et al., and Van Allen et al [25–27]. We annotated all variants using ANNOVAR since these mutation datasets lacked the annotation field required for GVB calculation [84].

We also used 10 transcriptomic datasets from GEO, which were GSE7553, GSE8401, GSE15605, GSE46517, GSE22153, GSE22154, GSE54467, GSE59455, GSE65904, and GSE81383, and one dataset from Tirosh et al. Most transcriptome datasets were downloaded with probe-level identifiers (ID_REF) rather than gene symbols. Therefore, we used the GSEA software (v.4.3.2) to collapse datasets based on the chip platform, suitable for each data type if collapsed data were not provided [85, 86]. Specifically, we used median_probe in collapsing mode for the probe set ≥ 1 gene. We established two meta-cohorts (Meta-PM and Meta-SV) by integrating five transcriptomic datasets, respectively, enabling robust external validation: Meta-PM was used for primary versus metastases sample comparison. On the other hand, Meta-SV was used for survival analysis. When creating meta-cohorts, we conducted batch correction with pyComBat after merging log_2_(x + 1) transformed datasets [87, 88].

We analyzed two single-cell RNA sequencing (scRNA-seq) datasets, Gerber et al. and Tirosh et al [32, 33]. Preprocessing for Gerber et al. involved filtering to exclude cells expressing fewer than 200 genes, more than 9,000 genes, or greater than 1% mitochondrial transcripts, as well as removing genes detected in fewer than 20 cells. Processing for Tirosh et al. involved filtering to exclude cells expressing fewer than 200 genes, more than 11,000 genes, or greater than 60% mitochondrial transcripts, as well as removing genes detected in fewer than 3 cells. Counts were then normalized to a total of 10,000 transcripts per cell, followed by a log_1p_ transformation. We identified highly variable genes using standard parameters (minimum mean expression of 0.0125, maximum mean expression of 3, and minimum dispersion of 0.5). The untransformed counts were retained for regression steps. We regressed out the effects of total counts and mitochondrial content, then scaled all features to unit variance.

### External validation of SAM pairs in cBioPortal genomic datasets

We performed external validation of SAM pairs using somatic mutation and clinical data from three independent melanoma cohorts in cBioPortal [25–27]. We evaluated whether the SAM score, based on the co-impairment of SAM pairs, could stratify patients to survival prognosis in the independent datasets. We applied the same process and method used in the internal TCGA validation. After calculating the GVB and SAM scores, we divided the patients into two groups based on the average SAM score for each dataset: SAM-H^g^ and SAM-L^g^. Due to limited clinical variables in external datasets, we included as many overlapping clinical covariates as possible in the survival model. Therefore, for Liu et al., we used clinical information that includes age, sex, and survival information, OS (months), or PFS (months). For Snyder et al., we used clinical information, which includes sex and OS (months). For Van Allen et al., we used clinical information, which includes age, sex, and survival information, OS (months), or DFS (months). We used the SAM-L^g^ group as the reference group. The penalizer value was set to 0.01 to prevent convergence issues during the survival model fitting with Cox proportional hazards regression analysis in all datasets.

### External validation of SAM pairs in GEO meta-cohorts

We evaluated the SAM pairs to determine whether they could predict sample types and patients’ survival outcomes in external transcriptomic datasets by using the SAM score. In external validation for transcriptomic datasets, we used two meta-cohorts, Meta-PM and Meta-SV: 

We constructed Meta-SV using GSE22153, GSE22154, GSE54467, GSE59455, and TCGA-SKCM, which have clinical information related to survival analysis. We calculated the SAM score for each sample to determine whether both genes were inactivated. We stratified the samples into two groups: SAM-H^t^ and SAM-L^t^. The SAM-L^t^ group was used as a reference group in survival analysis. Since the clinical information of Meta-SV were insufficient compared to the TCGA-SKCM, we used information that overlapped with the clinical information used for survival analysis in TCGA-SKCM. As a result, we used age at initial diagnosis, sex, and OS (weeks) to survival analysis. However, the time unit of the OS each of the five datasets differed: days for GSE22153 and TCGA-SKCM, weeks for GSE22154, months for GSE54467, and weeks for GSE59455. Therefore, we normalized all OS values to weeks for consistency within Meta-SV. The penalizer value was set to 0.01 to prevent the convergence issue. 

We also constructed Meta-PM using GSE7553, GSE8401, GSE15605, GSE46517, and GSE65904, which have tumor sample type information, including primary and metastases for melanoma. We calculated the gene activation-based SAM score for each tumor sample. We compared the SAM scores between primary and metastases tumor samples in Meta-PM using a two-sided Student's *t*-test. Furthermore, we demonstrated that the SAM score could predict tumor sample types accurately using two-sided Fisher's exact test and calculating AUROC, AUPRC, accuracy, F1, precision, and recall. 

### Immune cell infiltration analysis

We conducted immune cell infiltration analysis on Meta-PM and Meta-SV using xCell [31]. We compared xCell scores between primary and metastases samples from Meta-PM and between SAM-H^t^ and SAM-L^t^ from Meta-PM and Meta-SV using the two-sided Student's *t*-test.

### Single-cell RNA sequencing analysis

We analyzed 92 scRNA-seq samples from Gerber et al. (GSE81383) and 737 samples from Tirosh et al., which have cell type information from CIBERSORTx [32, 33, 89]. We used the scanpy package in Python and preconfigured pipelines from Heumos et al [90, 91]. We performed principal component analysis on the scaled and normalized data. Next, we selected the number of principal components by identifying the elbow in the variance-explained curve. To define cell clusters, we used a k-nearest neighbor graph optimized by maximizing the silhouette score over a grid search and the Leiden algorithm. To annotate clusters, we examined marker gene expression using dot plots and mapped these to known immune cell types. In addition, we used a UMAP to visualize cell clustering and annotations. We also conducted a Chi-square test to determine whether samples stratified based on SAM score are related to the annotated group or malignant state. We performed the same processes except for cell annotation analysis to Tirosh et al.

### Functional enrichment analysis

We conducted an enrichment analysis by using a hypergeometric test from GSEApy to identify which protein assemblies from NeST are associated with the genes in SAM pairs [34, 35]. We considered assemblies with FDR < 0.1 to be significantly enriched and *P*-value < 0.05 to be enriched (FDR-BH adjustment). We also used single-sample gene set enrichment analysis (ssGSEA) from GSEApy to examine differential activation of pathways between the groups [34]. Therefore, we compared normalized ssGSEA scores between primary and metastases samples from Meta-PM and SAM-H^t^ and SAM-L^t^ from Meta-PM and Meta-SV.

### Machine learning approach to predict primary and metastases tumor samples

To evaluate whether genes in SAM pairs are predictive of melanoma metastasis, we used a machine learning (ML) framework using a balanced random forest (BRF) classifier from the imbalanced-learn Python package [92]. All transcriptomic datasets used in the ML framework were standardized using StandardScaler from Scikit-learn [88]. In within- and across-study predictions, we used Meta-PM for training data, which had primary and metastases tumor sample type information. As in the comparison of SAM scores analysis, we only used samples that had tumor sample type information. To obtain more precise prediction results, we used LOOCV and implemented it with the LeaveOneOut function of the Scikit-learn package in within-study prediction [87]. In the across-study prediction, we tested the trained model on Meta-SV. We selected expression features corresponding to genes in SAM pairs or genes associated with melanoma metastasis previously reported in 11 independent studies (Supplementary Data S2, S9) [11, 42–51]. Only genes present in both training and test sets were used.

We used metrics area under the receiver-operating characteristic curve (AUROC), area under the precision-recall curve (AUPRC), accuracy, F1, precision, and recall to evaluate the models’ predictive performances in within-study prediction. To quantify the prediction results of ML models, we defined true positive (TP) as the case where an actual metastases tumor sample is predicted as a metastases sample. We also defined true negative (TN) as the case where an actual primary tumor sample is predicted as a primary sample. In contrast, a false positive (FP) was defined as the case where a primary tumor sample was predicted as a metastasis sample. Moreover, a false negative (FN) was defined as the case where a metastases tumor sample was predicted as a primary sample. The metric calculation formula for measuring the performance of each model is as follows:

13 $$\:AUROC=\:{\int\:}_{0}^{1}TPR\left({FPR}^{-1}\right(x\left)\right)dx$$

14 $$\:AUPRC=\:{\int\:}_{-\infty\:}^{\infty\:}Prec\left(c\right)dP\:(Y\le\:c)$$

15 $$\:Accuracy=\:\frac{TP+TN}{TP+FN+TN+FP}$$

16 $$\:F1=\:\frac{2}{(\frac{1}{Precision}+\frac{1}{Recall})\:}$$

17 $$\:Precision=\:\frac{TP}{TP+FP}$$

18 $$\:Recall=\:\frac{TP}{TP+FN}$$

We also used hazard ratio (HR) and *P*-value from Cox proportional hazards regression analysis as a metric to evaluate the models’ predictive performances from across-study predictions for Meta-SV. We used predicted primary samples as a reference group. The penalizer value was set to 0.01 to ensure model stability.

We used the SHapley Additive explanation (SHAP) to identify important predictive features in within- and across-study predictions [52]. We calculated the mean (|SHAP values|) for each gene in SAM pairs from the within- and across-study models trained on Meta-PM.

To ensure the robustness and reliability of the predictive performance of the ML model constructed by genes in SAM pairs with balanced random forest classifier, we used Monte-Carlo cross-validation 1,000 times with a randomly split dataset with 90% for the training set and 10% for the test set [93]. We used a two-sided Student’s *t*-test to compare the models’ predictive performance.

### Drug repurposing to identify candidate anti-metastasis drugs for melanoma

To identify potential anti-metastasis drugs for melanoma, we constructed a drug repurposing pipeline based on expression signatures derived from genes in SAM pairs, network proximity, and literature evidence.

The drug repurposing progress with expression signatures is as follows: (i). We obtained expression signatures of genes in SAM pairs from the Meta-PM through differential gene expression (DGE) analysis by using the R package limma [94]. When conducting DGE analysis, we set the primary samples as the control group and the metastases samples as the test group. (ii). We extracted the expression information (Gene symbol and log_2_(FC)) of the genes in SAM pairs from the DGE results of the Meta-PM as the melanoma metastasis-related expression signature to iLINCS (http://ilincs.com) [24]. Specifically, we selected genes with FDR < 0.1 to define SAM-based metastasis-related signatures (FDR-BH adjustment). After submitting the SAM signatures to iLINCS, (iii). from disease-related signatures, we validated the submitted SAM gene expression signatures by confirming a significantly positive Pearson correlation with melanoma-related disease states. (iv). From cancer therapeutics response signatures and pharmacogenomics transcriptional signatures, we identified candidate anti-metastasis drugs for melanoma as those showing a significantly negative Pearson correlation between their expression profiles and the SAM signature within skin cancer tissues or cell lines.

We conducted network analyses to identify the relationship between the target genes of candidate anti-metastasis drugs for melanoma and genes belonging to SAM pairs or common melanoma and neoplasm metastasis-related genes in the PPI network. Therefore, we demonstrated whether genes in SAM pairs could form a non-random, modular network by calculating the largest connected component (LCC) of genes in SAM pairs to compare it to the relative LCC of random gene sets of equal size in the PPI network. To demonstrate that genes in SAM pairs could have efficacy against melanoma metastasis with network analyses, we calculated the network proximity between the genes in SAM pairs and 2 common genes associated with both melanoma and metastasis from The Comparative Toxicogenomics Database (CTD) and BioPlanet from Enrichr [34, 95]. We also performed the same network proximity analysis between target genes of candidate compounds and (i). genes in SAM pairs or (ii) 2 common genes associated with both melanoma and metastasis, whether the candidate compound could have anti-metastatic efficacy for melanoma.

The network proximity, which is *d(S, T)*, was defined by Gueny et al. as follows:

19 $$\:d(S,\:T)\:=\:\frac{1}{\parallel\:T\parallel\:}\sum_{t\in\:T}\:{min}_{s\in\:T}d(s,\:t)$$

The *d(S, T)* is the shortest path length between nodes *S* and *T* in the network. *S* represented genes in the SAM pairs or genes associated with both melanoma and metastasis. *T* represented the individual drug target genes. We used the toolbox package of Gueny et al. (https://github.com/emreg00/toolbox) to calculate the largest connected component and network proximity [58]. We downloaded the v.12.0 human PPI network from STRING DB [59]. Particularly, we used only PPIs with an interaction score greater than 700 to use a high-confidence PPI network in the analyses [96]. 

 Finally, we manually reviewed previously published studies to identify whether the candidate compounds have been reported to reduce melanoma metastasis risk.

## Supplementary Information

Supplementary material 1.

Supplementary material 2.

## Data Availability

Variant summary data were downloaded from ClinVar (https://www.ncbi.nlm.nih.gov/clinvar/). pLOF data metrics by gene were downloaded from gnomAD (https://gnomad.broadinstitute.org/). Somatic mutation data from the TCGA MC3 project, clinical data from TCGA CDR, and mRNA expression data were downloaded from the Pan-Cancer Atlas (https://gdc.cancer.gov/about-data/publications/pancanatlas). Human experimentally or computationally discovered SL data were downloaded from the supplementary data of Lee et al. and SynLethDB (https://www.synlethdb.com/) [18, 81]. Gene essentiality profile data were downloaded from the supplementary data from Nichols et al [82]. Somatic mutation and clinical datasets of Liu et al., Snyder et al., and Van Allen et al. were downloaded from cBioPortal (https://www.cbioportal.org/). NeST assemblies were downloaded from the supplementary data of Kong et al [97]. The hierarchy of NeST was downloaded from the Network Data Exchange (NDEx) [98]. The external gene expression and clinical data sets for melanoma, including survival or tumor sample information, were obtained from the GEO repository (https://www.ncbi.nlm.nih.gov/geo/) with the accession codes GSE22153, GSE22154, GSE54467, GSE59455, GSE7553, GSE8401, GSE15605, GSE46517, GSE65904, and GSE81383. The 737 single-cell RNA sequencing data of Tirosh et al. were downloaded from CIBERSORTx (https://cibersortx.stanford.edu/) [89]. The STRING PPI network v.12.0 was downloaded from the STRING website (https://string-db.org). The drug target information used to discover anti-metastasis drugs was obtained from the Cancer Therapeutics Response Portal v2 (https://portals.broadinstitute.org/ctrp.v2/) [61]. The pathway information and melanoma-related genes were obtained from GO biological process, Reactome, Elsevier pathway collection, and BioPlanet from Enrichr (https://maayanlab.cloud/Enrichr/) [95]. The melanoma, cutaneous malignant and neoplasm metastasis-related genes were obtained from the CTD database (https://ctdbase.org/) [60]. All data used in this study are publicly available.

## Abbreviations

AUROC: Area under the receiver-operating characteristic curve
AUPRC: Area under the precision-recall curve
BRF: Balanced random forest classifier
CTD: Comparative toxicogenomics database
DEG: Differentially expressed gene
DFI: Disease-free interval
DGE: Differential gene expression
DSS: Disease-specific survival
FC: Fold change
FDR: False discovery rate
GEO: Gene expression omnibus
GN: Gaussian naïve bayes classifier
GVB: Gene-wise variant burden
HR: Hazard ratio
KNN: K-nearest neighbor classifier
LOOCV: Leave-one-out cross-validation
LR: Logistic regression classifier
ML: Machine learning
OS: Overall survival
RF: Random forest classifier
SVC: Support vector machine classifier
PCA: Principal component analysis
PFI: Progression-free interval
PPI: Protein-protein interaction
SIFT: Sorting intolerant from tolerant
SKCM: Skin cutaneous melanoma
