# Supplementary material for: Mutation-informed gene pairs to predict melanoma metastasis
Source: Cell Commun Signal. 2026 Jan 29;24:130. doi: 10.1186/s12964-025-02602-4 (PMC12924559; doi:10.1186/s12964-025-02602-4)
Supplement: Supplementary file 2 — Supplementary material 2. [file 12964_2025_2602_MOESM2_ESM.docx]

**Supplementary Information**

**Title: Mutation-informed gene pairs to predict melanoma metastasis**

To whom correspondence should be addressed:

Ju Han Kim, M.D., Ph.D.

Seoul National University Biomedical Informatics (SNUBI), Department of Biomedical Sciences, Seoul National University College of Medicine, Seoul, Republic of Korea

Email: [juhan@snu.ac.kr](mailto:juhan@snu.ac.kr); Tel: +82-2-740-8320; Fax: +82-2-3673-2167

**Table of contents**

The supplementary Information file contains:

4 Supplementary Figures, and 12 Supplementary Tables

**Supplementary Figure 1. Assessing the predictive power of gene-wise variant burden (GVB) scores in the TCGA-SKCM dataset.** **A** Density of GVB scores, but excluding when GVB is 1 of the SKCM patients. **B** Density of all GVB scores of SKCM patients.

**Supplementary Figure 2. Single-cell RNA sequencing analysis for Tirosh et al. and Gerber et al.**

**A** UMAP of the 737 cells of Tirosh et al., colored according to cell type annotated by Tirosh et al. **B, C** UMAP of the 92 cells of Gerber et al., colored according to (**B**) groups annotated by Gerber et al. and (**C**) clusters annotated by analysis using scanpy. **D** Dot plot showing the expression of marker genes of each identified cluster in 92 cells from Gerber et al. **E** Cell type annotation to each cluster visualized by UMAP for Gerber et al.

**Supplementary Figure 3. Prediction performances comparison using Monte-Carlo cross-validation in within-study prediction.**

**A** Box plots represent the predictive performances of an ML model constructed by expression profiles of genes in SAM pairs using various ML methods based on Monte-Carlo cross-validation (1,000 times). **B** Box plots represent the predictive performances of ML models constructed by expression profiles of various melanoma metastasis-related biomarkers based on Monte-Carlo cross-validation (1,000 times). Statistical significance was measured by a two-sided Student’s *t*-test (*P-value < 0.05, **P-value < 0.01, and ***P-value < 0.001). Boxplot shows the median value, outliers, and interquartile range (IQR) as bounds of the box with whiskers extending to the upper and lower quartiles ± 1.5 times the IQR.

**Supplementary Figure 4. Network characteristics of genes in SAM pairs.**

**A** The size of the largest connected component (LCC) of genes in SAM pairs in the PPI network. **B** Network proximity between genes in SAM pairs and genes associated with both melanoma and metastasis in the PPI network. Statistical significance of the size of the LCC, the network proximity was measured by the empirical distribution.

| **Dataset** | **Cancer type** | **Patient** | **Gene** | **Mutation** |
| --- | --- | --- | --- | --- |
| Liu et al | SKCM | 144 | 15,810 | 86,725 |
| Snyder et al | SKCM | 64 | 11.972 | 35,838 |
| Van Allen et al | SKCM | 110 | 13,814 | 52.988 |

**Supplementary Table 1. Description of the cBioPortal dataset.**

This table shows the external somatic mutation and clinical data that we used to perform external validation (survival analysis) on the SAM pairs discovered from TCGA-SKCM.

| **Dataset** | **Sample** | |
| --- | --- | --- |
|  | **Have all clinical features** | **Have insufficient clinical features** |
| GSE22513 | 54 | 3 |
| GSE22154 | 22 | 0 |
| GSE54467 | 79 | 0 |
| GSE59455 | 122 | 19 |
| TCGA-SKCM | 455 | 15 |
| Total | 732 | 37 |

**Supplementary Table 2. Description of the GEO dataset used in survival analysis.**

This table shows the external transcriptomic data used when performing external validation to construct Meta-SV (survival). We performed survival analysis using samples that had all clinical features.

| **Dataset** | **Sample** | | |
| --- | --- | --- | --- |
|  | **Primary** | **Metastases** | **Unclassified** |
| GSE7553 | 14 | 40 | 33 |
| GSE8401 | 31 | 52 | 0 |
| GSE15605 | 46 | 12 | 16 |
| GSE46517 | 31 | 73 | 17 |
| GSE65904 | 16 | 188 | 10 |
| Total | 138 | 365 | 76 |

**Supplementary Table 3. Description of the GEO dataset used in the analysis to compare the SAM score.**

This table shows the external transcriptomic data used when performing external validation to construct Meta-PM (primary and metastases). We only analyzed primary and metastases samples to compare the SAM scores.

|  | **Group 1** | **Group 2** | **Group 3** |
| --- | --- | --- | --- |
| SAM-H^t^ | 17 | 15 | 16 |
| SAM-L^t^ | 25 | 10 | 9 |
| Total | 42 | 25 | 25 |

χ^2^ = 4.32, *df* = 2, P-value = 0.115

**Supplementary Table 4. Presentation of results for 92 melanoma samples grouped by SAM score to each group in Gerber et al.**

*df*, degree of freedom.

|  | **B cells** | **CAF** | **Endothelial cells** | **Macrophages** | **NK cells** | **T cells CD4** | **T cells CD8** | **Malignant** |
| --- | --- | --- | --- | --- | --- | --- | --- | --- |
| SAM-H^t^ | 42 | 8 | 24 | 20 | 9 | 138 | 61 | 85 |
| SAM-L^t^ | 23 | 17 | 5 | 20 | 4 | 71 | 53 | 157 |
| Total | 65 | 25 | 29 | 40 | 13 | 209 | 114 | 242 |

χ^2^ = 64.93, *df* = 7, P-value = 1.551 x 10^-11^

**Supplementary Table 5. Presentation of results for 737 melanoma samples grouped by SAM score to each cell type in Tirosh et al.**

*df*, degree of freedom.

|  | **B1 B** | **CD16+ Mono** | **Erythroblast** | **G/M prog** | **Lymph prog** | **NK** |
| --- | --- | --- | --- | --- | --- | --- |
| SAM-H^t^ | 6 | 7 | 11 | 8 | 3 | 13 |
| SAM-L^t^ | 7 | 7 | 16 | 7 | 2 | 4 |
| Total | 13 | 14 | 27 | 15 | 5 | 17 |

χ^2^ = 5.777, *df* = 5, P-value = 0.329

**Supplementary Table 6. Presentation of results for 92 melanoma samples grouped by SAM score to each cell type in Gerber et al.**

*df*, degree of freedom.

| **Model** | **# of biomarkers in the dataset** | **HR** | **95% CI** | **P-value** |
| --- | --- | --- | --- | --- |
| BRF | 106 | 0.721 | 0.600-0.868 | 0.0005 |
| GN | 106 | 0.737 | 0.584-0.929 | 0.009 |
| KNN | 106 | 1.097 | 0.685-1.756 | 0.7 |
| LR | 106 | 0.84 | 0.666-1.058 | 0.138 |
| RF | 106 | 1.334 | 0.997-1.785 | 0.053 |
| SVC | 106 | 0.938 | 0.759-1.16 | 0.556 |

**Supplementary Table 7. Multivariate Cox proportional hazards regression of overall survival across-study predicted primary samples: comparison across various ML methods.**

Genes in SAM pairs were used for across-study prediction in feature selection, trained by Meta-PM, and tested for Meta-SV. P < 0.05 was considered statistically significant.

HR, Hazard Ratio; CI, Confidence Interval.

| **Biomarker** | **# of biomarkers in the dataset** | **HR** | **95% CI** | **P-value** |
| --- | --- | --- | --- | --- |
| B Song et al | 7 | 1.448 | 1.202-1.735 | 9.75E-05 |
| B Huang et al | 4 | 0.762 | 0.632-0.919 | 0.004 |
| G Jia et al | 2 | 0.856 | 0.709-1.033 | 0.105 |
| H Luan et al | 4 | 1.107 | 0.921-1.330 | 0.281 |
| L Zhang et al | 2 | 1.013 | 0.840-1.222 | 0.891 |
| MK Leonard et al | 4 | 0.926 | 0.767-1.118 | 0.423 |
| R Huang et al | 1 | 1.007 | 0.834-1.217 | 0.937 |
| SAM genes | 106 | 0.721 | 0.600-0.868 | 0.0005 |
| S Bhalla et al | 3 | 0.987 | 0.821-1.19 | 0.894 |
| X Wu et al | 2 | 1.3 | 1.079-1.565 | 0.0058 |
| X Zhou et al | 3 | 0.965 | 0.803-1.160 | 0.704 |
| Y Nurzat et al | 3 | 1.191 | 0.990-1.433 | 0.063 |

**Supplementary Table 8. Multivariate Cox proportional hazards regression of overall survival across-study predicted primary samples: comparison across biomarker panels.**

Metastatic melanoma-associated biomarkers from previous studies and genes in SAM pairs were used across-study prediction in feature selection, trained by Meta-PM, and tested for Meta-SV. P < 0.05 was considered statistically significant.

HR, Hazard Ratio; CI, Confidence Interval.

| **Compound** | **# of genes** | **Pearson** | **P-value** |
| --- | --- | --- | --- |
| Avicin-D | 65 | 0.479691 | 0.000053 |
| (S)-Tipifarnib | 65 | 0.408961 | 0.000719 |
| CHM-1 | 65 | 0.395874 | 0.001098 |
| BRD-A05715709 | 65 | 0.393782 | 0.001173 |
| BRD-K02251932 | 65 | 0.392896 | 0.001206 |
| DBeQ | 65 | 0.374872 | 0.002093 |
| BRD-K48477130 | 65 | 0.363968 | 0.002879 |
| MLS002473913 | 65 | 0.342562 | 0.005218 |
| Alisertib | 65 | 0.312793 | 0.011184 |
| BRD-A28746609 | 65 | 0.304262 | 0.013733 |
| fluvastatin | 65 | 0.29203 | 0.018253 |
| KW-2449 | 65 | 0.258733 | 0.037427 |
| KI8751 | 65 | 0.255263 | 0.04015 |
| SGX-523 | 65 | 0.217099 | 0.082364 |
| PG 490 | 65 | -0.197943 | 0.113972 |
| Pifithrin | 65 | -0.232007 | 0.062933 |
| BRD-K37390332 | 65 | -0.24437 | 0.049786 |
| MLN 2480 | 65 | -0.254565 | 0.040718 |
| BRD-K13999467 | 65 | -0.25927 | 0.037019 |
| Tacedinaline | 65 | -0.266973 | 0.031569 |
| Brivanib | 65 | -0.272316 | 0.028197 |
| CAY10603 | 65 | -0.291546 | 0.018455 |
| Trametinib | 65 | -0.293842 | 0.017512 |
| CIL55 | 65 | -0.300681 | 0.014944 |
| MLS006011051 | 65 | -0.313048 | 0.011115 |
| RO4929097 | 65 | -0.322754 | 0.008736 |
| 2-(Allylamino)-3-chloronaphthoquinone | 65 | -0.322843 | 0.008716 |
| Pifithrin MU | 65 | -0.344963 | 0.004891 |
| BRD-K11533227 | 65 | -0.38494 | 0.001544 |
| PF-03758309 | 65 | -0.385724 | 0.001507 |
| SJ 172550 | 65 | -0.398015 | 0.001026 |
| PCI-34051 | 65 | -0.42329 | 0.000443 |

**Supplementary Table 9. Presentation of results for expression profile correlation of genes in SAM pairs between Meta-PM and compounds from iLINCS, CTRS library.** Results using data from cancer therapeutics response signatures (CTRS) provided one signature (specific cell line, concentration, and time) per compound.

| **Compound** | **# of genes** | **Pearson** | **P-value** |
| --- | --- | --- | --- |
| Erlotinib | 63 | 0.56116 | 0.000002 |
| Cisplatin | 63 | 0.462038 | 0.000138 |
| Dasatinib | 63 | 0.399675 | 0.001174 |
| Geldanamycin | 63 | 0.360623 | 0.003691 |
| Lapatinib | 63 | 0.14699 | 0.250311 |
| Doxorubicin | 63 | -0.027712 | 0.829304 |
| Gemcitabine | 63 | -0.145368 | 0.255634 |
| Topotecan | 63 | -0.234374 | 0.064476 |
| Bortezomib | 63 | -0.293806 | 0.019431 |
| Sirolimus | 63 | -0.33783 | 0.006772 |
| Vorinostat | 63 | -0.347152 | 0.005311 |
| Sorafenib | 63 | -0.367954 | 0.003008 |
| Sunitinib | 63 | -0.386699 | 0.001744 |
| 5-Azacytidine | 63 | -0.4333 | 0.000389 |

**Supplementary Table 10. Presentation of results for expression profile correlation of genes in SAM pairs between Meta-PM and compounds from iLINCS, PG library.** Results using data from pharmacogenomics transcriptional signatures (PG) provided multiple signatures (cell lines, concentrations, and times) per compound. Therefore, we collapsed multiple probes per gene by the mean to calculate correlation.

| **Library** | **Compound** | **Proximiry (d)** | **Proximity (z)** | **P-value** |
| --- | --- | --- | --- | --- |
| CTRS | DbeQ | 2 | -0.063969908 | 0.827 |
| CTRS | SGX-523 | 2 | -0.310595546 | 0.713 |
| CTRS | CAY10603 | 2 | -0.537246269 | 0.623 |
| CTRS | KW-2449 | 1 | -0.718245841 | 0.513 |
| CTRS | PCI-34051 | 2 | -0.822190465 | 0.487 |
| CTRS | BRD-K11533227 | 1.5 | -0.882410812 | 0.347 |
| CTRS | PF-03758309 | 2 | -1.275864199 | 0.321 |
| CTRS | KI8751 | 1.666666667 | -1.214201481 | 0.194 |
| CTRS | Tacedinaline | 1.8 | -1.330376276 | 0.18 |
| CTRS | BRD-A05715709 | 1 | -2.397341843 | 0.048 |
| CTRS | Pifithrin MU | 1.75 | -2.057139537 | 0.048 |
| CTRS | SJ 172550 | 1 | -2.171469828 | 0.045 |
| CTRS | RO4929097 | 2 | -2.49291358 | 0.015 |
| CTRS | Alisertib | 1 | -2.950473776 | 0.01 |
| CTRS | MLN 2480 | 0.666666667 | -4.879079195 | 0 |
| CTRS | Trametinib | 1 | -3.84705496 | 0 |
| PG | Sirolimus | 2 | -0.093372539 | 0.811 |
| PG | Brivanib | 2 | 0.444504948 | 0.788 |
| PG | Doxorubicin | 2 | -0.363577218 | 0.713 |
| PG | Gemcitabine | 2.333333333 | -0.444571908 | 0.52 |
| PG | Geldanamycin | 1 | -1.461041766 | 0.276 |
| PG | Vorinostat | 1.8 | -1.330376276 | 0.18 |
| PG | Bortezomib | 2 | -1.421704713 | 0.137 |
| PG | 5-Azacytidine | 1 | -1.989198438 | 0.102 |
| PG | Dasatinib | 1.8 | -1.625830789 | 0.092 |
| PG | Sunitinib | 1.666666667 | -1.927926384 | 0.048 |
| PG | Erlotinib | 1 | -2.270325083 | 0.044 |
| PG | Lapatinib | 1 | -2.270325083 | 0.044 |
| PG | Topotecan | 1 | -2.727755356 | 0.028 |
| PG | Sorafenib | 1 | -3.922465925 | 0.001 |

**Supplementary Table 11. Presentation of results for network proximity between compounds’ target genes and common biomarkers of melanoma and neoplasm metastasis.**

CTRS, Cancer therapeutics response signatures; PG, Pharmacogenomics transcriptional signatures

| **Library** | **Compound** | **Proximiry (d)** | **Proximity (z)** | **P-value** |
| --- | --- | --- | --- | --- |
| CTRS | Trifluoperazine | 2 | 1.114000654 | 1 |
| CTRS | Tacedinaline | 1.4 | 0.92579389 | 0.927 |
| CTRS | BRD-K11533227 | 1 | -0.275092049 | 0.889 |
| CTRS | DBeQ | 1 | -0.406818345 | 0.858 |
| CTRS | SJ 172550 | 1 | -0.389699333 | 0.83 |
| CTRS | SGX-523 | 1 | -0.671847311 | 0.689 |
| CTRS | CAY10603 | 1 | -0.623100158 | 0.678 |
| CTRS | BRD-A05715709 | 1 | -0.741606987 | 0.619 |
| CTRS | Alisertib | 1 | -0.748915275 | 0.592 |
| CTRS | KI8751 | 1 | -0.833377334 | 0.515 |
| CTRS | KW-2449 | 1 | -0.95260688 | 0.454 |
| CTRS | PF-03758309 | 1 | -1.246775275 | 0.355 |
| CTRS | RO4929097 | 1 | -2.616138459 | 0.018 |
| CTRS | MLN 2480 | 0.666666667 | -2.546608534 | 0.015 |
| CTRS | Tramenitib | 0.5 | -2.685608448 | 0.012 |
| CTRS | Pifithrin MU | 0.75 | -2.679314939 | 0.011 |
| PG | Bortezomib | 2 | 2.51408958 | 1 |
| PG | Gemcitabine | 2 | 1.79382287 | 0.998 |
| PG | Geldanamycin | 1 | -0.141264483 | 0.962 |
| PG | Vorinostat | 1.4 | 0.92579389 | 0.927 |
| PG | Brivanib | 1.5 | 0.648445977 | 0.915 |
| PG | Erlotinib | 1 | -0.229719435 | 0.891 |
| PG | Lapatinib | 1 | -0.229719435 | 0.891 |
| PG | Sirolimus | 1 | -0.487950036 | 0.796 |
| PG | Doxorubicin | 1 | -0.569651921 | 0.755 |
| PG | 5-Azacytidine | 1 | -0.667148188 | 0.692 |
| PG | Dasatinib | 1.2 | -0.507044285 | 0.513 |
| PG | Topotecan | 1 | -0.984125976 | 0.508 |
| PG | Sunitinib | 1.166666667 | -0.52243742 | 0.499 |
| PG | Sorafenib | 0.75 | -2.289549073 | 0.015 |

**Supplementary Table 12. Presentation of results for network proximity between compounds’ target genes and genes in SAM pairs.**

CTRS, Cancer therapeutics response signatures; PG, Pharmacogenomics transcriptional signatures
